# Preliminary Exploration of Weekly Peer Group Discussions as a Strategy for Coping with Feelings Associated with Euthanasia in Dairy Caretakers

**DOI:** 10.3390/ijerph19042177

**Published:** 2022-02-15

**Authors:** Lily Edwards-Callaway, Hailey Simpson, Noa Román-Muñiz, Catie Cramer, Sage Mijares, Lorann Stallones, Jorge Rivera-Gonzalez, Jennifer Aberle

**Affiliations:** 1 Department of Animal Science, Colorado State University, Fort Collins, CO 80523, USA; hailey.simpson@colostate.edu (H.S.); noa.roman-muniz@colostate.edu (N.R.-M.); catie.cramer@colostate.edu (C.C.); sage.mijares@rams.colostate.edu (S.M.); jorge.rivera-gonzalez@colostate.edu (J.R.-G.); 2 Department of Psychology, Colorado State University, Fort Collins, CO 80523, USA; lorann.stallones@colostate.edu; 3 Department of Human Development and Family Studies, Colorado State University, Fort Collins, CO 80523, USA; jennifer.aberle@colostate.edu

**Keywords:** bereavement, caretakers, coping strategy, dairy cattle, euthanasia, grief

## Abstract

Dairy caretakers experience a variety of occupational risks including stress related to performing euthanasia and making euthanasia-related decisions for cattle in their care. Few supportive interventions exist to help caretakers cope with euthanasia-related stress. The aim of this study was to assess the impact of weekly peer discussion sessions as an intervention to reduce euthanasia-related stress and grief in dairy caretakers. This study utilized scores from a modified pet-based bereavement questionnaire to assess the change in bereavement of caretakers in response to euthanasia-related stress in a non-treatment group (who did not attend peer discussion sessions, *n* = 7) and a treatment group (who attended peer discussion sessions, *n* = 15). Key findings of this study were that discussion sessions did not have a direct impact on the study outcomes as measured using a pet bereavement scale, as there was no difference in the change in bereavement scores during the 8 week study period between the treatment and non-treatment groups. Thematic analysis of peer discussions revealed that compassion towards dairy cattle is a prominent factor in areas of decision making, protocols, and training. Further studies should continue to explore how performing euthanasia and making euthanasia-related decisions impacts caretakers and what supportive interventions can reduce stress and grief.

## 1. Introduction

Dairy farming today relies heavily on hired labor to provide animal care and harvest milk. As with other animal agricultural industries, foreign-born Latino workers make up a significant portion of the United States dairy farm labor force [1]. Animal caretakers on dairy operations experience a variety of stressors due to factors inherent to dairy production, daily job tasks, and work organization. Animal handling and machinery hazards, heavy workloads and long work shifts in variable and sometimes extreme environmental conditions have been reported by dairy workers as sources of stress [2,3,4]. In 2014, dairy cattle production accounted for almost half of all fatal occupational injuries in animal agriculture and aquaculture and over two-thirds of all cattle and ranching occupational fatal injuries [5]. Foreign-born workers have historically suffered a greater burden of occupational injuries than their native counterparts [6]. Despite the high number of occupational dairy worker injuries, previous work has highlighted their limited awareness of occupational risks [3,7], available medical and mental health resources [4,7], and their perception that work injuries are not preventable [3,7]. This environment, coupled with language barriers and lack of health insurance [8], plus inconsistent and limited training programs [2,3,4], makes these dairy workers a vulnerable population. Furthermore, psychosocial factors such as relationships with management and the quality of communication with management have been identified as predictors of perceived job stress for dairy workers [7].

Another potential source of job stress for dairy caretakers is performing euthanasia and making euthanasia-related decisions regarding the cattle they care for. Euthanasia is a critical management tool utilized across animal industries providing caretakers with a means to end animal suffering. Despite understanding that euthanasia may be the most humane choice for an animal, electing to and performing euthanasia can cause animal caretakers to experience “moral stress” [9], as they work daily to keep animals alive and well yet are also faced with making euthanasia decisions. Although caretakers recognize the important role of euthanasia in managing animal welfare, euthanasia decision making continues to be identified by caretakers across animal industries as a source of emotional stress, causing feelings of failure [10], frustration [4], sadness [11,12], and anger [12,13], among other similar sentiments. 

In the dairy industry, the timeliness of euthanasia has been identified as an area of concern and needed improvement due to negative animal welfare implications of poor decision making [14,15,16]. Although there are many factors impacting end-of-life decisions on dairies, the feelings that caretakers have for cows and calves have been identified as significant contributors to how decisions are made [15]. Regardless of the type of animal industry, making objective end-of-life decisions can be a challenge for caretakers and owners who have a strong emotional investment and responsibility to help the animals under their care [4,9,10,11,12,13].

In companion animal owners, the psychological impact of bereavement, i.e., losing a loved pet, and the subsequent grief and stress have been studied extensively [17,18,19,20,21,22,23,24,25]. Research with employees in animal shelters, veterinary clinics, and biomedical research facilities has indicated that employees involved in performing euthanasia as part of their job showed significantly greater levels of work-related stress, stress-induced physical ailments, depression and unresolved grief reactions, work–family conflict, burnout, turnover, and dissatisfaction with their work [12,26,27,28]. In a study of animal shelter workers, participants indicated that counseling, job rotation, more job assistance, breaks and time off, support groups, meetings, better communication, skill-based trainings, stress and coping seminars, and employee appreciation and morale-boosting initiatives were potential support strategies that could help them cope with euthanasia-related stress [29].

Bereavement associated with the loss of production animals has not been previously studied but recent research indicates that dairy workers care deeply about the cattle they care for and that, although they recognize the importance of ending animal suffering, making the decision to euthanize cattle is a source of work-related stress [4]. Focus groups facilitated on dairies in Colorado revealed that although participants mentioned several ways in which they deal with the stress of performing euthanasia, caretaker knowledge of emotional-support services to help cope with stress was limited to non-existent [4]. Additionally, there is little available information regarding effective and culturally responsive support strategies that could be implemented into employee training and wellness programs on dairy farms to help employees cope with the euthanasia-related stress and resulting grief. Therefore, understanding the feelings experienced by dairy caretakers associated with the loss of euthanized cattle could be helpful in establishing supportive interventions to help with the euthanasia-related stress experienced by dairy caretakers.

Based on the clear need for developing coping strategies for dairy workers who experience euthanasia-related stress and grief, this pilot study aimed to assess the impact of weekly peer discussion sessions as a supportive intervention to reduce euthanasia-related stress and grief in dairy workers. Additionally, a modified pet-based bereavement questionnaire was used to assess the degree of and change in bereavement of dairy caretakers in response to facing euthanasia of dairy cows and calves under their care. Authors hypothesize that after a series of peer discussion sessions, dairy caretakers will report lower bereavement and caretakers will find peer discussions a helpful coping mechanism for euthanasia-related grief.

## 2. Materials and Methods

**Figure 1:**
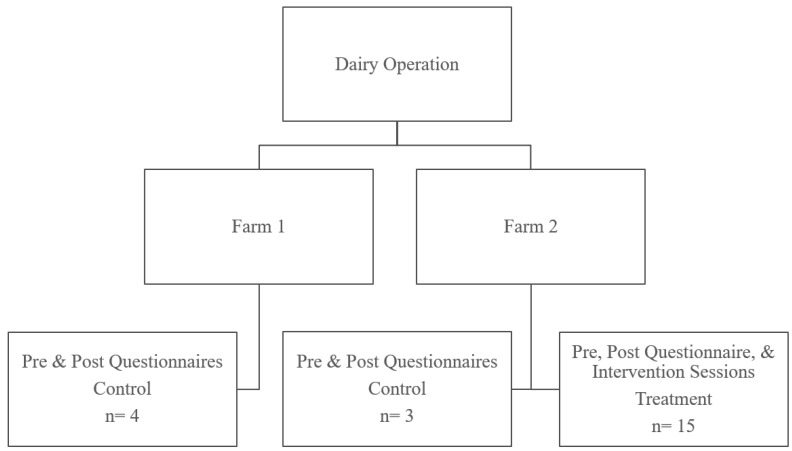
Flowchart identifying study population and treatment allocation. It is common for one dairy operation to have multiple farm sites.

This study was approved by the Colorado State University Institutional Review Board (#20-10315H) prior to project initiation. The intent of this study was to test the effect of weekly peer discussion groups on bereavement of dairy workers who perform cattle euthanasia by comparing the change in bereavement scores, as measured using a pre- and post-questionnaire, between a non-treatment group (no intervention) and a treatment group (weekly peer discussion groups focused on euthanasia-related issues). This study was conducted from November 2020 to January 2021. A project flowchart is shown in Figure 1.

### 2.1. Study Population and Recruitment

The population of interest for this study was dairy caretakers who perform euthanasia as a routine part of their job. Participants were recruited from two dairy sites (Farm 1 and Farm 2) within the same dairy operation. The non-treatment group consisted of caretakers who worked at Farm 1 with both cows and calves and in the calf yards at Farm 2 (Figure 1). The treatment group consisted of caretakers from Farm 2 only that had little interaction with the other study participants (Figure 1). This treatment allocation was used to ensure that discussions about the project between groups that could have impacted the effects of the treatment were minimized. The participating dairy operation was progressive and engaged in the areas of worker and animal health and well-being.

Prior to study initiation, researchers obtained verbal consent from all participating individuals. The non-treatment group participants (*n* = 7) were offered a $15 gift card for completing both the pre- and post-questionnaires for this study. The treatment group participants (*n* = 15) received differing gift card denominations depending on the number of weekly sessions they attended (6 sessions or more = $45, 4 or 5 sessions = $30) and only if they completed both pre- and post-questionnaires.

### 2.2. Weekly Peer Discussion Sessions

#### 2.2.1. Treatment Group

Individuals from Farm 2 were recruited to take part in peer discussion sessions that occurred once weekly over an 8 week period. Attendance varied from week to week, but a core group of attendees participated in at least six of the eight discussions. Supervisors and workers met in separate groups. Discussions were held in Spanish to accommodate the preferred language of the participants and were led by four different bilingual facilitators, who were all proficient in Spanish. Each discussion session consisted of the facilitator asking approximately four open-ended questions (e.g., How do family, friends, coworkers, or bosses respond to your feelings about euthanasia? What strategies for coping do you find helpful? What has someone said or done to help you when performing or making euthanasia decisions?) related to euthanasia, which differed from week to week. The format of the discussions and the questions asked were adapted from an adult bereavement support group model [30] and revised to be relevant to dairy caretakers. This format was chosen as previous research with caretakers from other animal industries experiencing loss has suggested that support groups, meetings, and overall communication and discussion may be helpful and supportive strategies [29,31,32,33,34]. Refreshments were provided at each meeting to create a welcoming environment. All discussion groups were recorded using the Voice Memos application on an iPad (Apple Inc., Cupertino, CA, USA), conducted at the end of a dairy shift, and held in a private meeting room on the farm.

#### 2.2.2. Non-Treatment Group

The non-treatment group did not participate in weekly discussion sessions during the 8 week period.

### 2.3. Questionnaires

All study participants, including both non-treatment and treatment groups, were asked to fill out questionnaires at the beginning (pre-questionnaire) and completion (post-questionnaire) of the 8 week study period. The pre-questionnaire consisted of 20 demographic questions, general questions about euthanasia on the farm (e.g., euthanasia methods and frequency), and 22 Likert scale questions (Supplementary Materials). Sixteen of the questions came directly from a pet bereavement scale developed and validated by Hunt and Padilla [35] adjusted to be relevant to dairy caretakers. The Hunt and Padilla [35] questionnaire was originally developed as an instrument to measure the psychological impact of losing a pet and documented three distinct factors including grief, anger, and guilt. Though the survey in this research was adjusted to be relevant to dairy caretakers and included questions about cattle instead of pets, the questions were still related to the 3 factors of bereavement: grief, anger, and guilt. Post-questionnaires included the same questions as the pre-questionnaires except demographic and general euthanasia questions were removed.

Questionnaires were available in both English and Spanish and administered in person. The treatment group completed their pre-questionnaires on the first day of sessions and non-treatment groups completed their pre-questionnaires during the same week. To maintain confidentiality of responses, participants were assigned an alphanumeric code which was used to track session attendance and match pre- and post-questionnaire responses. Participants were given a post-questionnaire at the end of the 8 week study. Treatment group participants completed their post-questionnaires after their last peer discussion group and non-treatment group participants completed their post-questionnaires within the same week.

### 2.4. Data Analysis

#### 2.4.1. Coding

All audio recordings of sessions were transcribed and then translated into English by one researcher (JRG). Researchers did not record which individuals spoke during the sessions. Methods described by Braun and Clarke [36] were followed for thematic analysis of peer-group discussion transcripts. Seven researchers from the core research team reviewed all transcripts and identified initial themes. Three of the researchers then independently coded transcripts for these defined themes. Validation of coding was achieved by discussion of differences among coders. After discussion, agreement was reached for each coded transcript. Each coder contributed different lenses of understanding to coding which helped develop a rich understanding of the material recorded from the weekly sessions. One coder considered herself a dairy industry expert and insider after growing up on a dairy, earning her Doctor of Veterinary Medicine degree, and conducting extensive research and outreach work with Colorado’s dairy industry. A second coder also considered herself a dairy expert, as she has completed her Doctoral degree in Dairy Sciences and has delivered impactful research to the dairy industry specifically on dairy calf health and behavior. The third coder considered herself to be an industry outsider, and has earned her bachelor’s degree in animal science and continues to work on her Master’s research specifically on livestock behavior and welfare.

#### 2.4.2. Questionnaire Analysis

All participants elected to fill out the pre- and post-questionnaire in Spanish. Pre- and post-questionnaire data were manually entered into Microsoft Excel (Microsoft Corporation, Washington, DC, USA). Demographic information was summarized using descriptive statistics. Following the method of Hunt and Padilla [35], answers to the 16 Likert scale questions were assigned a numerical value (i.e., Strongly disagree = 0, Disagree = 1, Agree = 2, Strongly agree = 3). An overall bereavement score was calculated by summing the answers for the 16 questions for each individual participant. A lower score indicated a lower level of bereavement, while a higher score indicated a greater level of bereavement. Scores for each of the three subfactors were calculated by taking the average response to the questions within each subcategory (grief = 7 questions; anger = 5; guilt = 4). The change in overall bereavement and change in the three subfactors over the 8 week study period was calculated by subtracting the pre-questionnaire scores from the post-questionnaire scores. A Wilcoxon’s Rank Sum test was performed in SAS 9.4 (SAS, Cary, NC, USA) to assess differences in the pre-questionnaire median scores between the treatment and non-treatment groups to identify if pre-questionnaire bereavement scores were significantly different before the intervention was administered. Additionally, Wilcoxon’s Rank Sum tests (SAS 9.4, Cary, NC, USA) were used to determine differences in the change in overall bereavement and subfactor scores between the non-treatment and treatment groups.

## 3. Results

### 3.1. Weekly Peer Discussion Sessions

There were 15 treatment participants (*n* = 7 supervisors, *n* = 8 workers). Weekly peer discussion sessions lasted on average 11:04 min (range 6:46–14:39 min). A total of 16 sessions were held (8 for workers and 8 for supervisors) over the 8 week study period. It is important to note that though there was a total of 15 participants in the peer discussion sessions, attendance varied weekly. The number of participants at each session can be found in Table 1.

#### Themes

Coauthors identified eight themes from a preliminary review of the session transcripts: Training, Protocols, Animal Welfare and Compassion, Attitudes towards Euthanasia, Decision-Making, Internal and External Communication, Coping Strategies, and Areas for Improvement Related to Euthanasia. Definitions of these themes, main concepts identified and examples of quotes representing each theme can be found in Table 2. There were several instances in which two themes were coded for certain transcript excerpts. The Animal Welfare and Compassion theme commonly occurred with the Attitudes towards Euthanasia theme. Participants’ attitudes towards euthanasia often included feeling compassion towards the cows they euthanized. When participants expressed how they felt having to perform euthanasia, they expressed that they felt sad or frustrated but often commented that this was necessary in order to ensure animal welfare and stop animal suffering. Additionally, the Decision-Making theme and Animal Welfare and Compassion theme commonly occurred together. It was apparent that participants made euthanasia decisions based on their compassion for the animals they cared for.

### 3.2. Questionnaires

#### 3.2.1. Demographics

A total of 14 matching pre- and post-questionnaires were used for analysis (6 non-treatment and 8 treatment participants). Five participants only completed the pre-questionnaire and thus were removed from the analysis. Three participants selected the same answer, the first option, for all Likert questions and were removed from the analysis as it was apparent that these participants did not answer individual questions. 

Of the 14 participants, 1 (7%) participant identified as female. A single participant (1, 7%) identified as non-Hispanic or Latino with their primary language being English (although this participant elected to complete the questionnaire and weekly sessions in Spanish) while all other participants (13, 93%) identified as Hispanic or Latino with their primarily language being Spanish. Participants were asked how long they had been working in their current position, to which 3 (21%) said they had worked in their current position for 6 months to 1 year, 4 (28%) said 1 to 2 years, and 7 (49%) said more than 2 years, but less than 10 years. 

All participants had been trained to perform euthanasia and used a penetrating captive bolt gun followed by potassium chloride injection as the primary method of euthanasia. Depending on dairy protocols and an individual’s job description, a caretaker may make decisions about when to euthanize an animal, may only perform the act of euthanasia, or could be responsible for both. Participants were asked how often they make the decision to euthanize cattle; three participants (21%) said they make euthanasia decisions daily. Participants were asked how often they perform euthanasia on farm, to which 7 (49%) said they perform euthanasia weekly, and 7 (49%) said they perform euthanasia monthly. 

#### 3.2.2. Bereavement Scores

**Figure 2:**
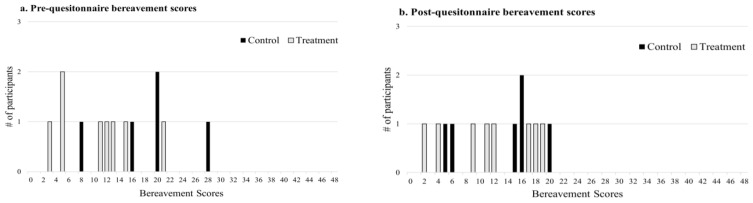
(**a**,**b**) Frequency of overall bereavement scores from pre- and post-questionnaires of the non-treatment (*n* = 6) and treatment groups (*n* = 8).

Figure 2a,b shows the frequencies of overall bereavement scores from pre- and post-questionnaires for non-treatment and treatment groups. Per the Hunt and Padilla [35] calculation for overall bereavement score, participants could have scored between a 0 and a 48. In the present study, bereavement scores were relatively low for both pre- and post-scores. The overall bereavement scores in this study ranged from 3 to 28. The median scores for all bereavement outcomes for the non-treatment and treatment groups for both questionnaires are reported in Table 3. There was no difference in the pre-questionnaire overall bereavement scores between the non-treatment and treatment groups (*p* = 0.092).

Table 4 shows the median change between pre- and post-questionnaire scores for overall bereavement and all three subfactors. There was no significant difference in the change of any of the scores between the treatment and non-treatment group (*p* ≥ 0.135). 

## 4. Discussion

Resources to support dairy caretakers in coping with work-related emotional stress, such as that resulting from performing euthanasia, are limited. Research in other animal industries, including shelter and companion animal medicine, have identified the importance of providing outlets for individuals to cope with having to perform euthanasia as part of their job [12,26,29]. The intent of this pilot study was to implement weekly peer discussion sessions as a potential supportive strategy to provide dairy caretakers with an outlet to deal with grief and other feelings associated with end-of-life decision making and performing euthanasia on the dairy. Although the sessions did not have a direct impact on the study outcomes as measured using a pet loss-based bereavement scale, valuable information was gleaned that provides a foundation for future research in this critical area.

The participants in this study were representative of the Western United States dairy industry workforce. In the current study, most participants (93%, 13) identified as Hispanic or Latino, spoke Spanish (86%, 12) as their primary language, and identified as men (93%, 13). These demographics are consistent with those reported by previous studies conducted with dairy caretakers in the Western United States [3,4,7]. The average age of participants in this study was 32 years which is younger than the national average(57.5 years; [37]). The use of weekly peer discussions was a culturally-appropriate intervention for dairy workers as it aligned with best practices previously identified for health and safety training programs for Latino/a dairy caretakers [3]. Specifically, the weekly peer discussion in the present study sought to understand and involve workers using community-based participatory methods, which brings together coworkers to learn together and build trust [3,38]; the weekly peer discussions offered an opportunity for dairy workers to share their perspectives with researchers and coworkers. Weekly peer discussions further aligned with best practices for Latino/a dairy caretakers as the discussions were language and literacy appropriate and promoted active participation by asking a variety of questions and encouraging all participants to share [3].

During weekly discussion sessions, participants expressed feelings of compassion for the dairy cattle they cared for and demonstrated a drive to ensure animal well-being by alleviating animal suffering. They recognized that euthanasia can be a mechanism for alleviating suffering even if losing the animal also made them feel sad. The theme of Animal Welfare and Compassion regularly was connected with comments related to Decision-Making, identifying how the desire to ensure animal welfare was a driving force in euthanasia decision-making. A study by Román-Muñiz et al. [4] also reported that dairy caretakers who participated in focus groups about perspectives on euthanasia demonstrated compassion for the cattle they cared for, indicating that cattle deserved kindness and that euthanasia was a way to do this. In the current study, the theme of Animal Welfare and Compassion was intertwined with many other topics, demonstrating how these two elements were prominent factors in areas of decision-making, protocols, and training. Similarly, Wagner et al. [10] reported that dairy producers in focus groups regarding perspectives on euthanasia emphasized improving animal welfare and cited the occurrence of compassion fatigue and emotional stress as challenges. Participants in the treatment group of this study also expressed feelings of frustration, sadness, and sometimes impotence from not being able to prevent injury or illness in the dairy cattle they cared for. These feelings expressed during the weekly sessions may explain, in part, the suggestions for more learning opportunities and prevention strategies to assist caretakers in making better treatment and/or euthanasia decisions proactively as a mechanism to alleviate animal suffering sooner.

The application of a pet loss-based bereavement questionnaire in this study was a unique approach to assessing the response of dairy caretakers to the loss of the animals that they work with. Bereavement is the experience of recent loss of a significant person due to death [39]. The feelings following loss of a beloved pet due to death mirror that of human loss [18,19,20,21,23] although it should be acknowledged that there are challenges in comparing loss experiences due to the complexity of factors impacting bereavement in addition to study methodologies. The impacts of bereavement manifest in a multitude of feelings often dominated by grief that often have negative physical, emotional, and psychosocial impacts on the affected individuals [9,20,24,25]. Hunt and Padilla [35] created a pet loss-based bereavement scale as a means of having a standardized approach to identifying at-risk individuals for application in research and clinical intervention. The scale aimed to assess the degree of three feelings commonly identified by bereaved individuals, grief, anger, and guilt, some of which may be relevant to dairy caretakers experiencing stress related to performing euthanasia. In the current study, focus group questions were written so that participants could share freely how they felt in their own words. Within the theme of Attitudes towards Euthanasia, feelings of sadness, frustration, and powerlessness were mentioned by participants, identifying perhaps some different emotional responses to euthanasia than those captured within the bereavement questionnaire. Although evidence exists regarding dairy workers’ compassion towards cattle [4], it is likely that the feelings experienced by dairy workers after euthanizing a cow are not the same as an owner who loses their pet due to known differences in the role of each type of animal. As described, there is a plethora of work exploring the reactions of pet owners to the loss of a pet but to the authors’ knowledge there is no research that exists asking dairy caretakers about their relationships with the animals they care for. As such, more research is needed to better quantify the experience of dairy caretakers after euthanizing an animal specifically related to loss so that the effect of interventions can be quantified. 

In the current study, there was no significant difference in the change in bereavement score and subfactor scores during the 8 week study period between the treatment and non-treatment groups. Generally, the overall bereavement scores observed in this study were relatively low as compared with those measured in Hunt and Padilla [35]. The greatest bereavement score possible using the Hunt and Padilla [35] scale is 48; comparatively, the range of scores in the current study was from 3 to 28, suggestive of a low degree of bereavement experienced by the dairy caretakers. Hunt and Padilla [35] reported an overall mean bereavement score of 28 (SD = 8.6) in their study of companion animal pet owners who recently suffered the loss of a pet, with 50% of the study participants scoring between 23 and 35. Hunt and Padilla [35] concluded, based on their distribution of sub-factor scores, that grief may stand apart from the other sub-factors of guilt and anger and that most bereaved pet owners experience greater levels of grief compared to the other sub-factors. The sample size in the current study is relatively low and therefore it is a challenge to make conclusions about the distribution of subfactor scores. What is noteworthy in the current study is that the median subfactor scores for grief for both non-treatment and treatment groups (pre-questionnaire: 1.3 and 0.65; post-questionnaire: 0.75 and 0.65, respectively) were considerably lower than that observed in the Hunt and Padilla [35] study (median = 2.6). Bereavement is potentially not the most appropriate measurement to use when exploring feelings of livestock caretakers as suggested in these preliminary findings. Although more work needs to be done in this area, these preliminary results provide some initial illustration of the feelings dairy caretakers experience in the context of loss as it is evaluated in pet owners.

It is worth noting that the pre-questionnaire median bereavement score of the non-treatment group (18) was numerically greater than that of the treatment group (11.5) at the start of this study; the difference was not significant (*p* = 0.092) but the authors feel this is an important point for consideration and future work. Due to the nature of how treatment groups were assigned to prevent cross-communication between groups, all of the non-treatment group participants worked with calves, half of them worked exclusively with calves. Authors speculate that feelings associated with the suffering, loss, or euthanasia of calves may be different than that felt when working with cows. Recent work conducted by our research group demonstrated that dairy caretakers found caring for sick calves to be especially distressful and caretakers worked diligently to care for sick calves [40]. In a study of shelter caretakers, participants reported that euthanasia felt like a beneficial act and authors speculated that this may have been particularly salient in old animals [12] suggestive of the fact that euthanizing younger animals can be more emotionally distressing. Additionally, in dairy systems in North America “surplus calves,” which are animals that are not going to be used to replace the milking herd, are often euthanized, likely driven by a lack of a market for the calves [41,42]. This creates a unique scenario in which caretakers may be expected to euthanize healthy, newborn animals potentially exacerbating emotional distress in caretakers. While this is not a likely situation for the dairy caretakers in the current study as they do not euthanize surplus calves, exploring the differing impacts of euthanasia and loss for dairy caretakers that care for calves, as compared with cows, is an area that deserves further investigation.

Although dairy caretakers feel a bond between themselves and the cows and calves they care for, the level of attachment between caretakers and cattle may be different than that found between a pet and an owner. Interestingly, in focus groups with dairy caretakers discussing perspectives of euthanasia, the participants likened caring for cattle to humans caring for pets in the way they are taken care of when sick and euthanized if that was the best way to alleviate animal suffering [4]. There is limited to no research explaining the nature of the attachment between caretakers and dairy cattle but research in companion animals has demonstrated that there is positive relationship between attachment and/or closeness with the deceased and subsequent grief [19,22,43,44,45,46]. This would be an interesting area to explore further as understanding the nature of the human-animal bond in the context of production animal caretakers could help in the development and refinement of supportive strategies.

There are many factors in addition to attachment that are predictive of the emotional distress experienced during bereavement including demographic factors such as gender [35,47,48,49], age [35,43], ethnicity [44,50], and social network [23,43,46]. As discussed previously, the population of dairy caretakers in the United States is predominantly Spanish-speaking Latino men. Conversely, in many of the studies focused on pet bereavement and grief, the study populations are predominantly non-Hispanic women [17,19,21,23,35,47,48,51,52]. Multiple studies have identified that women experience and/or cope with grief differently and often experience greater levels or intensities of despair and grief than men [43,44,47,48]. The current study only included one woman participant so perhaps the effect of weekly peer discussions on bereavement would have been different with a more gender diverse population. The impact of ethnicity has been minimally studied [44,50] as an impact factor in bereavement studies but is a critical factor to explore when trying to create and identify culturally responsive intervention strategies for dairy caretakers. Studies have also explored the impacts of age on grief and coping and reported varying results [17,35,43,53]; age can be a challenging indicator to evaluate across studies as it is often categorized and considered differently in analyses. Hunt and Padilla [35] reported that older individuals felt less guilty about the death of their pet than younger study participants, speculating that this could be due to several factors including acceptance that death of a pet is inevitable. Although the average age of the current study participants was relatively young compared to other aforementioned studies, perhaps the participants’ experience with having to make euthanasia decisions helped them accept euthanasia as an inevitable and sometimes essential component of animal care, similar to individuals who have had several pets over their lifetimes. Swine caretakers and veterinarians have indicated in previous survey studies that performing and making decisions about euthanasia gets easier with more experience [54,55]. Perhaps job experience could be explored in future studies as another factor impacting feelings associated with the loss of production animals due to euthanasia. It should be noted that some studies have explored numerous additional factors (e.g., age of pet, cause of death) and also identified relationships between factors (e.g., pet attachment and age), therefore it is essential to evaluate how various factors impact bereavement holistically.

Currently, formalized interventions such as the sessions implemented in this study are not a part of human resource support services on most dairy operations (personal communication, I.N. Román-Muñiz). These sessions could well fit into the regular meeting schedule that dairy operations hold with their employees. Similar to how progressive operations discuss worker safety and preventive strategies against work-related injuries and illnesses, feelings around euthanasia decisions and process could be regularly discussed. Previous work in the occupational safety and health space has identified interventions that utilize collective discussions are a way to engage and empower workers [3]. The normalization of the topic of euthanasia has been suggested as a way to improve awareness and healthcare seeking among dairy caretakers [4] and regular discussions similar to the current intervention could serve that purpose. The support strategy of peer discussion groups was based off interventions that have been successful in other applications. Animal shelter workers indicated that strategies such as support groups, meetings and better communication may be helpful in dealing with euthanasia-related stress in the workplace [29]. Additionally, some pet owners experiencing grief from having lost a pet have explained that simply talking about losing their pets made them feel better [31]. Veterinarians often play a role in providing support to bereaved pet owners, which includes providing recommendations on coping strategies which often includes support groups [32,33,34]. From a broader perspective, social networks have been shown to be influential in how individuals cope with the negative feelings associated with bereavement [23,43,46], thus support groups serve a role to provide community and a supportive social network to those in need.

Despite there being no difference in study outcomes related to caretaker feelings, the thematic analysis revealed some valuable feedback from participants on the intervention. Participants indicated that the discussions gave them increased confidence in their decision-making as they saw that there was agreement between team members. When asked to provide feedback on the structure of the sessions, participants indicated that it would have been helpful to include a reflection portion of the session to discuss case studies to help them prepare for future euthanasia scenarios. Additionally, suggestions for improvement included more diverse questions and the presence of more co-workers, as attendance was sometimes limited to a core group. The sessions were relatively short on average and it is the authors’ perception that perhaps once a week was too frequent for these types of meetings; holding meetings perhaps once a month would provide more opportunity to share thoughts and events that had occurred over a longer period of time thus creating opportunity for a robust discussion. Additionally, the sessions were held at the end of shift and perhaps participating individuals were eager to finish their workday and return home. Outside of the workplace, participants also indicated a variety of coping strategies they used to help deal with work-related stress including talking to friends or family, spending time with pets, listening to music, talking to peers, or spending time alone. 

With a small sample size limited to one dairy, the conclusions that can be drawn from this feedback are limited. The participating dairy is a progressive dairy that is engaged in employee well-being; the culture of this particular dairy could be such that the team already works well together and perhaps the weekly meetings did not add the same level of value that maybe they would have on a different dairy. It is critical as future projects are developed to include a wide variety of dairies in order to capture how different farm cultures will respond to these types of interventions.

Throughout the qualitative analysis, a desire for more learning opportunities was evident. The dairy caretakers in this study population demonstrated a desire to improve their euthanasia decisions and suggested additional learning opportunities to enhance their skills. Other studies exploring perspectives of livestock caretakers on euthanasia have identified a similar desire by individuals making these end-of-life decisions for more training, with an emphasis on in-person, on-the-job experiences [3,56,57]. In this study, it was clear through focus groups that training and protocols served an integral part in euthanasia decision-making. Future work could explore the development of innovative training methods that both provide new technical knowledge to caretakers while also enhancing critical thinking skills, providing a platform for sharing ideas, and ultimately fostering a supportive work community.

## 5. Conclusions

Although the weekly peer discussion sessions did not change the level of bereavement of participants, the sessions allowed participants to share their feelings and ideas with each other. Despite there being no statistical difference in bereavement scores between treatment and non-treatment groups, thematic analysis provided further evidence that dairy caretakers have compassion for the dairy cattle they care for and that euthanasia is complex as it alleviates animal suffering but that it is an emotionally distressful decision to make and act to perform. Interestingly, workers commented that regular discussion sessions increased their confidence in making euthanasia decisions, suggesting that regular discussions regarding complex topics like euthanasia should be integrated into staff meetings on dairies. Additionally, the results indicated a relatively low level of bereavement when using a scale adapted from pet bereavement, highlighting two important concepts: the bond between dairy caretakers and cattle may differ from the bond pet owners feel towards their pets and more work is needed to develop and validate a tool to better measure bereavement in dairy caretakers. Findings from this study support previous work which indicates dairy caretakers have compassion for the cattle they care for and they recognize that euthanasia is a necessary means to end animal suffering. However, making the decision to euthanize is difficult given the competing emotions. Therefore, there is a need to identify appropriate strategies to support dairy caretakers during the euthanasia decision-making process and afterwards to support potential feelings of bereavement. Future studies should include novel approaches to both training and support strategies to help caretakers make improved end-of-life decisions. Moving forward, it is important for the dairy industry to have available mental health resources in order to help their caretakes cope with the loss of euthanized cattle. The resources must be culturally responsive and offered in ways that reduce stigma associated with seeking this kind of help.

## Figures and Tables

**Table 1:** Number of participants in each discussion session for both workers and supervisors, who met separately.

	Number of Participants Week							
Group	1	2	3	4	5	6	7	8
Worker	7	5	6	4	2	3	4	3
Supervisor	5	5	5	5	4	5	4	4

**Table 2:** Theme definitions, main concepts within each theme, and transcript excerpts for each theme.

Theme and Definition	Main Concepts	Primary Examples
**Training** Comments on how participants were trained, descriptions of training, and frequency of training for euthanasia	Outside of classroom training, caretakers received on the job training from peers. Participants, both workers and supervisors, agreed that training for euthanasia was sufficient.	“*The people who were already in the hospital, who had more experience, and had been already authorized. Because in the first case that I saw today, because they explained to me why, and how to do it, and what is the goal of doing it..*.” “*Yes, just as them, we are satisfied with the training they gave us. Because beside training us on how to perform euthanasia they also, we said why it is important to make the euthanasia decision. And so that’s why. I agree with the training we were given.*”
**Protocols** Comments about having or using euthanasia protocols	Euthanasia protocols were integral to decision making.	“*As one receives the animals, that you are in charge of, it is to take the most care and to comply with the protocol, let say in our case, that it is to check the pens so, to concentrate with what is being done to detect/diagnose the animals*.” “*Yes, the protocol we all have is the same procedure and that is why we take the same criteria, in all dairy farms for when making business decisions.*”
**Animal Welfare and Compassion** Comments revealing compassion towards cows, reducing suffering and pain	Applied specifically to feelings towards cattle and wanting to ensure their well-being. Associated with participants wanting to alleviate suffering.	“*Explaining truthfully that it is better to end the animal, than if the animal continues to lay there and kicking, suffering. And from that people do understand. People know what it is one’s responsibility to end the animal’s suffering.*” “*We let people know that animals must not suffer and that we must end the pain of animals*.”
**Attitudes towards Euthanasia** Comments about feelings related to euthanizing cows	Participants noted feeling sad, frustrated, and powerless associated with having to perform euthanasia. This theme occurred regularly with Animal Welfare and Compassion theme.	*“One feels sad because it is a life, but unfortunately, so that the animal no longer suffers, it has to be done.*” *“Well, first of all, we are human beings and although we are aware that the animal is going to stop suffering but, it is difficult to stop feeling like pity, sadness or affection for the animal that is going to be euthanized.*”
**Decision-Making** Comments related to how euthanasia decisions are made on farm	Trainings, protocols, and experiences helped participants make euthanasia decisions. Participants were generally in agreement on euthanasia decisions.	“*Well, if one of us makes the decision, it’s because we know it’s the best decision for the cow.*”
**Internal and External Communication** *(2 subthemes)*		
*Internal Communication * Comments about communication within the farm about euthanasia	Participants relied on communication between one another when it came to on-the-job training and making euthanasia decisions. Supervisors made final decisions on euthanasia cases but considered the opinions of other workers.	“*Yes, I think everyone is part of it, it is better to make the decision, they give me their opinions.*” “…*usually they are the ones who tell me that this cow must be euthanized, and I say yes or no*”
*External Communication* Comments about talking to the community, consumers and family members about the dairy industry and euthanasia on the farm	Most participants did not talk to family or friends about their euthanasia work. Participants felt it necessary to explain why euthanasia was important when given the opportunity.	“*It depends on how you explain it to the person, that is how they will understand it. If you put it in a bad way, they who don’t work with animals, will stay with that bad image. And that’s why it depends on how you talk about it.*”
**Coping Strategies** Comments describing dealing with euthanasia including detachment from work, support systems used to cope with euthanasia-related stress, and activities implemented as coping strategies	Work-specific support systems included discussing decisions with peers. Talking to friends or family, playing sports, listening to music, or spending time with animals were mentioned as ways to cope with euthanasia-related stress.	“W*ell you have to ask for support from your manager, so that he backs you up with some of the decisions and supports you.*”
**Areas for Improvement Related to Euthanasia** *(3 subthemes)*		
*Learning Opportunities * Comments about ideas for resources, experiences, and training that would help participants with performing euthanasia in the future	Participants wanted to improve to prevent the next euthanasia.	“*Yes, learning also “okay, what I could have done better” or what will do better for next time*.”
*Prevention Strategies * Strategies workers and supervisors use to prevent or better manage illness and injury to reduce the need to perform euthanasia	This often occurred when participants talked about wanting to improve the well-being of their cows and reduce suffering. Participants recognized that sometimes euthanasia was unavoidable.	“*A bit of impotence. For not having been able to do anything to prevent the animal from reaching euthanasia.*” “*At the end of the day, sometimes it is not in your hands to avoid euthanasia. For example, the cow may slip… um, how, how do you avoid that.”*
*Suggestions for Interventions * Potential benefits and areas for improvement for education and mental health interventions	Most recommendation occurred during the last discussion group. Participants suggested time to reflect after discussions, diversity in questions, having more staff present, and cases studies to learn from. Discussions increased confidence in participants as they saw agreeance amongst their peers on decision making.	*“That not everyone who did the euthanasia was here every day and if the whole group had been, every day. Maybe they had different experiences, different opinions to us.*” “*Oh, for me it was to hear that we are all on the same channel. That we all have the same criteria, perhaps for the same training we receive but, I think we are all on the same.*”

**Table 3:** The median overall and subfactor bereavement scores (grief, anger, and guilt) for pre- and post-questionnaires in both the non-treatment (*n* = 6) and treatment groups (*n* = 8) ^1^.

	Pre-Questionnaire Median (Lower, Upper Quartile)		Post-Questionnaire Median (Lower, Upper Quartile)	
	Non-Treatment	Treatment	Non-Treatment	Treatment
**Overall Score**	18 (13, 20)	11.5 (5, 14)	15.5 (6, 16)	11.5 (6.5, 17.5)
**Grief**	1.3 (1, 1.6)	0.65 (0.25, 1.2)	0.75 (0.3, 1.3)	0.65 (0.2, 1)
**Anger**	0.7 (0.6, 1)	0.3 (0.1, 0.7)	0.6 (0.4, 0.8)	0.5 (0.2, 1.1)
**Guilt**	1.13 (1, 1.5)	1 (0.5, 1.25)	1 (0.5, 1.25)	1 (0.63, 1.38)

^1^ Scores for analysis were calculated as in Hunt and Padilla [35]. The overall bereavement score was calculated by summing the answers for the 16 questions for each individual participant. Scores for each of the three subfactors were calculated by taking the average response to the questions within each subcategory (grief = 7 questions; anger = 5; guilt = 4). The change in overall bereavement and all four subfactors over the 8 week study period was calculated by subtracting the pre-questionnaire scores from the post-questionnaire scores.

**Table 4:** The median change in overall and factor bereavement scores (grief, anger, and guilt) between pre- and post-questionnaires in both the non-treatment (n = 6) and treatment groups (n = 8) ^1^.

	Change in Score Median (Lower, Upper Quartile)		*p*-Value
	Non-Treatment	Treatment	
**Overall Score**	−3.50 (−7.0, −1.0)	−1.50 (−3.50, 2.50)	0.241
**Grief**	−0.36 (−0.71, 0)	−0.14 (−0.57, 0.36)	0.642
**Anger**	−0.20 (−0.20, 0)	0 (−0.10, 0.30)	0.135
**Guilt**	−0.38 (−1.0, 0.25)	0 (−0.25, 0.38)	0.268

^1^ Scores for analysis were calculated as in Hunt and Padilla [35]. The overall bereavement score was calculated by summing the answers for the 16 questions for each individual participant. Scores for each of the three subfactors were calculated by taking the average response to the questions within each subcategory (grief = 7 questions; anger = 5; guilt = 4). The change in overall bereavement and all four subfactors over the 8 week study period was calculated by subtracting the pre-questionnaire scores from the post-questionnaire scores.

## Data Availability

Please contact the corresponding author with enquires about data availability.

## Supplementary Materials

The following supporting information can be downloaded at: https://www.mdpi.com/article/10.3390/ijerph19042177/s1, File S1: Questionnaire.
