# Supplementary material for: Preliminary Exploration of Weekly Peer Group Discussions as a Strategy for Coping with Feelings Associated with Euthanasia in Dairy Caretakers
Source: Int J Environ Res Public Health. 2022 Feb 15;19(4):2177. doi: 10.3390/ijerph19042177 (PMC8872095; doi:10.3390/ijerph19042177)
Supplement: Supplementary file 1 [file ijerph-19-02177-s001.zip › ijerph-1547340-supplementary.pdf]

File S1. Questionnaire

Participant ID: ___________

1. What gender do you identify with?
   _____ Male
   _____ Female
   _____ Other
   _____ Decline to answer

2. What ethnicity do you identify as?
   _____ Hispanic or Latino
   _____ Non-Hispanic or Latino
   _____ Decline to answer

3. What is your country of origin? _______________

4. What is your preferred language?
   _____ English
   _____ Spanish
   _____ Other (specify) _________________

5. How long have you been in your current position at work?
   _____ less than 6 months
   _____ 6 months to 1 year
   _____ 1-2 years
   _____ more than 2 years, but less than 10 years
   _____ 10 years or more

6. Have you worked on other dairies?
   _____ Yes
   _____ No

7. If yes, for how long?
   _____ less than 6 months
   _____ 6 months to 1 year
   _____ 1-2 years
   _____ more than 2 years, but less than 10 years
   _____ 10 years or more

8. What is your age (years)? ___________

9. What area do you work in on the dairy? (Select all that apply).
_____ maternity pen
_____ calf area
_____ hospital
_____ breeding
_____ feeding
_____ other (specify): _______________

10. What is your position on the dairy? (Select all that apply).
_____ caretaker/worker (cares for animals and does not oversee other people)
_____ supervisor (oversees one area on the dairy)
_____ manager (oversees multiple areas on the dairy)
_____ veterinarian
_____ other (specify): _______________

11. Have you been trained on how to perform euthanasia at your current place of work?
_____ yes
_____ no

12. Select the types of training that you received. (Select all that apply).
_____ in-person outside trainer
_____ in-person in-house trainer
_____ video
_____online
_____ written materials
_____ shadowing a co-worker
_____ other (specify): _______________

13. What methods of euthanasia are used on this farm to euthanize cows? (Select all that apply).

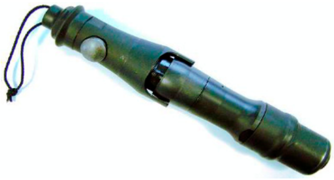

______ captive bolt

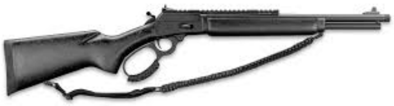

_____ gunshot

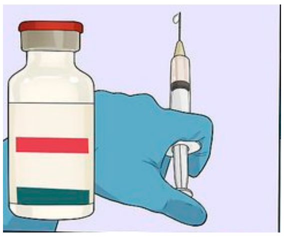

______ injectable (specify) __________________

______ other (specify)________________

14. What methods of euthanasia are used on this farm to euthanize calves? (Select all that apply).

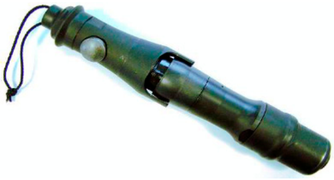

______ captive bolt

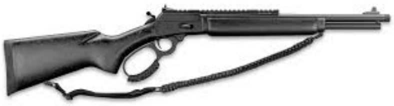

_____ gunshot

______ injectable (specify) _______________

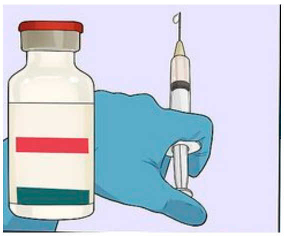

______ other (specify)________________

15. What is your preferred method of euthanasia and why?

16. What other methods of euthanasia have you used at other operations?

17. How often do you perform euthanasia on this farm?
    _____ daily
    _____ weekly
    _____ monthly
    _____ yearly
    _____ I do not perform euthanasia

18. How often do you make decisions about which animals need to be euthanized on this farm?
    _____ daily
    _____ weekly
    _____ monthly
    _____ yearly
    _____ I do not make decisions about euthanasia

Approximately how many cows have been euthanized on this farm in the past month?
_____ (number)
_____ I don’t know

Approximately how many calves have been euthanized on this farm in the past month?
_____ (number)
_____ I don’t know

In this questionnaire, “these cattle” refer to the cattle that have been euthanized on this farm. Cattle includes cows and calves.

The post-survey only included these questions.
Please rate the following items: Disagree Strongly, Disagree, Agree, Agree Strongly

1. I am very upset about the death of the cattle euthanized on this farm.

   Disagree Strongly Disagree Agree Agree Strongly

2. My life feels empty without these cattle.

   Disagree Strongly Disagree Agree Agree Strongly

3. I feel lonely without these cattle.

   Disagree Strongly Disagree Agree Agree Strongly

4. I miss these cattle enormously.

   Disagree Strongly Disagree Agree Agree Strongly

5. I cry when I think about these cattle.

   Disagree Strongly Disagree Agree Agree Strongly

6. I am sad about the death of these cattle.

   Disagree Strongly Disagree Agree Agree Strongly

7. I'll never get over the loss of these cattle.

   Disagree Strongly Disagree Agree Agree Strongly

8. I feel angry at the vet or farm management for not being able to save these cattle.

   Disagree Strongly Disagree Agree Agree Strongly

9. I feel angry at myself for not being able to save these cattle.

   Disagree Strongly Disagree Agree Agree Strongly

10. I have had nightmares about the cattle’s death.

Disagree Strongly Disagree Agree Agree Strongly

11. I am angry at other people for contributing to the death of these cattle.

Disagree Strongly Disagree Agree Agree Strongly

12. I have scary memories of the cattle’s illness/injury or death.

Disagree Strongly Disagree Agree Agree Strongly

13. I am angry at my friends/family for not being more helpful.

Disagree Strongly Disagree Agree Agree Strongly

14. I should have known that something bad could have happened to these cattle.

Disagree Strongly Disagree Agree Agree Strongly

15. I feel bad that I didn’t do more to save these cattle.

Disagree Strongly Disagree Agree Agree Strongly

16. I wish I had shown these cattle more kindness.

Disagree Strongly Disagree Agree Agree Strongly

17. Discussing my feelings on euthanasia with my co-workers makes me feel uncomfortable.

Disagree Strongly Disagree Agree Agree Strongly

18. Discussing my feelings on euthanasia with my co-workers makes me feel ashamed.

Disagree Strongly Disagree Agree Agree Strongly

19. Discussing my feelings on euthanasia with my co-workers makes me sleep better.

Disagree Strongly Disagree Agree Agree Strongly

20. Discussing my feelings on euthanasia with my co-workers helps me alleviate stress.

Disagree Strongly Disagree Agree Agree Strongly

21. Discussing my feelings on euthanasia with my co-workers makes me feel sad.

Disagree Strongly Disagree Agree Agree Strongly

22. Discussing my feelings on euthanasia with my co-workers makes me feel content.

Disagree Strongly Disagree Agree Agree Strongly
